# First Annual Meeting of the New England Dental Society

**Published:** 1883-11

**Authors:** 


### NEW ENGLAND DENTAL SOCIETY—FIRST ANNUAL MEETING AT PROVIDENCE, RHODE ISLAND.

The New England Dental Society (formerly the Merrimac Valley Dental Society), is the largest and oldest dental society in New England, having members from all sections of the New England States. It meets annually in different localities, and held its first annual meeting under its new organization, at Franklin Lyceum Hall, on Thursday and Friday, October 4 and 5, 1883. The following were the officers of the association:

President—Dr. Thos. Fillebrown, Portland, Me.; Vice Presidents—Dr. Wm. Barker, Providence; Dr. James Lewis, Burlington, Vt.; Secretary—Dr. A. M. Dudley, Salem, Mass.; Treasurer—Dr. G. A. Gerry, Lowell, Mass.; Librarian—Dr. G. F. Waters, Boston. There is also an Executive Committee of five.

There was a large number of members and visitors in attendance, and the discussions were earnest, instructive and enthusiastic. The principal interest of the meeting centered in the address of Prof. Garretson of Philadelphia, a sketch of which is appended. He did not give a clinic as was expected, because no patients presented themselves. He did not read a formal paper, but spoke for some time upon the subject of dentition. He said:

In all methods of instruction the teacher should endeavor to make the subject-matter as plain as possible, and to this end all unnecessary technical terms should be eliminated. We find that any subject, no matter how intricate and complex it may at first sight appear, by careful study and familiar observation may be made very simple and easy to be understood. If the assertion should be made that each individual possessed a jaw, it would not be questioned ; furthermore it is conceded that this jaw goes through a process of growth; that there was a time when it was only half the size that it presents at present, and a time further back when only half of that size, and so on until we come to the time when it had no existence whatever. So with the dentinal germ which we find perfected in the adult; there must have been a time when it had a beginning, and this we may call the primitive dental groove.

The same is true of the development of the tooth perfected in the adult ; it must have had a very simple beginning, and advanced through the different stages to perfection. Let us understand at the outset that the tooth is not a dermoid tissue; it is *sui generis ;* furthermore, the alveolar process is not a portion of the jaw; it is an entirely separate structure. Now, taking the jaw proper and representing it by a broad line, we discover upon it a little something which we designate as the tooth germ, and conclude that it will develop into a tooth structure. The jaw is covered over its surface with a mucous membrane, which is elastic, and as this germ developes it raises the mucous membrane along with it, until finally we have the germ enlarged to the size and shape of the future tooth, constituting the pulp, still covered with this mucous membrane. This membrane we will call the *tunica propria,* and it is separated from the dental pulp only by a lubricating surface. As we know that the pulp is much larger in young teeth than in those that are older, so we also know that in the germ the pulp occupies all the space which is reserved for the dentine. This begins to shrink away from the tunica propria, forming the dentine in a manner which we will not now stop to explain.

At the same time that this germ is enlarging and developing, other processes are going on around it. The tooth when erupted will need firm support, and so the bony process begins to form around this germ, still beneath the mucous membrane. As it gradually increases in height it raises up the mucous membrane in the same manner that the germ is doing, but so closely does this membrane hug the germ that it is not pulled away from it, but is doubled upon it, and as we called the first membrane the *tunica propria,* we will call this the *tunica reflexa.* Now we see the manner in which the enamel is formed ; the space between these two membranes, the tunica propria and tunica reflexa, is filled with a substance which, when operated upon by the forces of life, is brought to a high state of inflammation. The dentine is a sub-dermoid structure, but as the cells are extended through the tunica propria they are modified to the cylindrical rods of the enamel. The formation of the cermentum is essentially the same, except that as it is to be covered by a persistent membrane it is modified in structure, it being nourished on one side by the tunica propria, and on the other by the tunica reflexa.

Thus it is that the nourishment of the tooth is not entirely dependent upon the pulp; nay, more than this; the pulp and pericementum may both be dead, and still the tooth retain sufficient vitality to remain in the system without bad effect. But we must remember that while the periodontal membrane is one of double supply, it is not a double membrane.

Dr. Charles T. Terry, an American dentist residing in Milan, Italy, was introduced, and by his desire the Secretary read a paper, of which the following are the salient points : The writer had supposed American teeth to be a little the worst on earth ; but he had found the people of Italy, and particularly of Switzerland, to possess teeth far worse than our own as a whole. He was told by intelligent people of these countries that the condition of the teeth was owing to the air and water, but examination of the teeth of animals native to these countries disproved this theory utterly. Two extremes of diet operate disastrously upon teeth ; the very rich, and very poor and insufficient. The peasantry of Zurich drank much sour wine (like vinegar). This takes the phosphate of lime from the system and ruins the teeth. Floating the food into the stomach is the worst possible thing to do. In certain parts of Switzerland the teeth are so soft and crumbly that it is impossible to build upon them. In fact, they are the worst teeth in the world, though on the Mediterranean they are nearly as bad. As lemon culture advances in Italy the children eat the fruit and the acid ruins their teeth, owing to their eating too little food to keep the vital elements properly nourished. In San Remo and Zurich the writer saw not a set of actually sound teeth. Overwork, nervous excitement, &c., are claimed as causing decay in teeth ; yet the poorer classes already mentioned never overwork nor worry, and their teeth are in a very bad condition. Swiss dentists he had found, as a class, to be very intelligent.

Boston was decided upon as the place for the next annual meeting. Officers for the ensuing year were elected as follows:

President—Dr. William Barker.

First Vice-President—Dr. James Lewis, Burlington, Vt.

Second Vice-President—Dr. J. B. Coolidge, Boston.

Secretary—Dr. A. M. Dudley, Salem.

Treasurer—Dr. G. A. Gerry, Lowell.

Librarian and Microscopist—Dr. G. F. Waters, Boston.

Executive Committee—Drs. D. M. Clapp, Boston; T. Fillebrown, Portland; F. Searle, Springfield; S. G. Stearns, Lynn; R. R. Andrews, Cambridge.

